# NormExpression: An R Package to Normalize Gene Expression Data Using Evaluated Methods

**DOI:** 10.3389/fgene.2019.00400

**Published:** 2019-04-30

**Authors:** Zhenfeng Wu, Weixiang Liu, Xiufeng Jin, Haishuo Ji, Hua Wang, Gustavo Glusman, Max Robinson, Lin Liu, Jishou Ruan, Shan Gao

**Affiliations:** ^1^School of Mathematical Sciences, Nankai University, Tianjin, China; ^2^College of Life Sciences, Nankai University, Tianjin, China; ^3^School of Biomedical Engineering, Health Science Center, Shenzhen University, Shenzhen, China; ^4^Institute for Systems Biology, Washington, DC, United States

**Keywords:** gene expression, normalization, evaluation, R package, scRNA-seq

## Abstract

Data normalization is a crucial step in the gene expression analysis as it ensures the validity of its downstream analyses. Although many metrics have been designed to evaluate the existing normalization methods, different metrics or different datasets by the same metric yield inconsistent results, particularly for the single-cell RNA sequencing (scRNA-seq) data. The worst situations could be that one method evaluated as the best by one metric is evaluated as the poorest by another metric, or one method evaluated as the best using one dataset is evaluated as the poorest using another dataset. Here raises an open question: principles need to be established to guide the evaluation of normalization methods. In this study, we propose a principle that one normalization method evaluated as the best by one metric should also be evaluated as the best by another metric (the consistency of metrics) and one method evaluated as the best using scRNA-seq data should also be evaluated as the best using bulk RNA-seq data or microarray data (the consistency of datasets). Then, we designed a new metric named Area Under normalized CV threshold Curve (AUCVC) and applied it with another metric mSCC to evaluate 14 commonly used normalization methods using both scRNA-seq data and bulk RNA-seq data, satisfying the consistency of metrics and the consistency of datasets. Our findings paved the way to guide future studies in the normalization of gene expression data with its evaluation. The raw gene expression data, normalization methods, and evaluation metrics used in this study have been included in an R package named NormExpression. NormExpression provides a framework and a fast and simple way for researchers to select the best method for the normalization of their gene expression data based on the evaluation of different methods (particularly some data-driven methods or their own methods) in the principle of the consistency of metrics and the consistency of datasets.

## Introduction

Global gene expression analysis provides quantitative information about the population of RNA species in cells and tissues (Lovén et al., 2012). High-throughput technologies to measure global gene expression levels started with Serial Analysis of Gene Expression (SAGE) and are widely used with microarray and RNA-seq (Gao et al., 2014). Recently, single-cell RNA sequencing (scRNA-seq) has been used to simultaneously measure the expression levels of genes from a single cell, providing a higher resolution of cellular differences than what can be achieved by bulk RNA-seq, which can only produce an expression value for each gene by averaging its expression levels across a large population of cells (Gao, 2018). Raw gene expression data from these high-throughput technologies must be normalized to remove technical variation so that meaningful biological comparisons can be made. Data normalization is a crucial step in the gene expression analysis as it ensures the validity of its downstream analyses (Lovén et al., 2012). The differential expression analysis or the co-expression analysis using the same dataset could produce significant different genes using different data normalization methods. Although the significance of data normalization in the gene expression analysis has been demonstrated (Bullard et al., 2010), how to select a successful normalization method is still an open question, particularly for scRNA-seq data.

Basically, two classes of methods are available to normalize gene expression data using global normalization factors. They are the control-based normalization and the average-bulk normalization. The former class of methods assumes the total expression level summed over a pre-specified group of genes is approximately the same across all the samples. The latter class of methods assumes most genes are not significantly Differentially Expressed (DE) across all the samples. The control-based normalization often uses RNA from a group of internal control genes (e.g., housekeeping genes) or external spike-in RNA [e.g., ERCC RNA (Jiang et al., 2011)], while the average-bulk normalization is more commonly used for their universality. Five average-bulk normalization methods designed to normalize bulk RNA-seq data are library size, median of the ratios of observed counts that is also referred to as DESeq (Anders and Huber, 2010), Relative Log Expression (RLE), upper quartile (UQ), and Trimmed Mean of M values (TMM) (Robinson et al., 2010). Recently, three new methods were introduced as Total Ubiquitous (TU), Network Centrality Scaling (NCS), and Evolution Strategy (ES) with the best performance among 15 tested methods (Glusman et al., 2013). To improve scRNA-seq data normalization, Lun et al. (2016) introduced a new method using the pooled size factors (Pooled) and claimed that their method outperformed the library size method, DESeq and TMM. Bacher et al. (2017) addressed that using existing normalization methods on scRNA-seq data introduced artifacts that bias downstream analyses. Then, another new method SCnorm was introduced and claimed to outperform MR, Transcripts Per Million (TPM), scran, SCDE, and BASiCS using both simulated and case study data (Bacher et al., 2017).

Although many metrics have been designed to evaluate the relative success of these methods, different metrics or different datasets yield inconsistent evaluation results. Here raises another open question: principles need to be established to guide the evaluation of normalization methods. Glusman et al. (2013) proposed that a successful normalization method should simultaneously maximize the number of uniform genes and minimize the correlation between the expression profiles of gene pairs. Based on this criterion, they presented two novel and mutually independent metrics to evaluate 15 normalization methods and achieved consistent results using bulk RNA-seq data (Glusman et al., 2013). In this study, we designed a new metric named Area Under normalized CV threshold Curve (AUCVC) and applied it with another metric mSCC (see section “Materials and Methods”) to evaluate 14 commonly used normalization methods using both scRNA-seq and bulk RNA-seq data from the same library construction protocol. The evaluation results by both AUCVC and mSCC achieved consistency. In addition, the evaluation results using both scRNA-seq and bulk RNA-seq data also achieved consistency. So, we propose a principle that one normalization method evaluated as the best by one metric should also be evaluated as the best by another metric (the consistency of metrics) and one method evaluated as the best using one dataset should also be evaluated as the best using another dataset (the consistency of datasets). The datasets using different protocols (RNA-seq, scRNA-seq, or microarray) need to be used to validate the consistency, which is beyond the scope of this study. As many new normalization methods are being developed, researchers need a fast and simple way to evaluate different methods, particularly some data-driven methods or their own methods, rather than obtain information from published evaluation results, which could have biases or mistakes, e.g., misunderstanding of RLE, UQ and TMM (see section “Results”). To satisfy this demand, we developed an R package NormExpression including the raw gene expression data, normalization methods and evaluation metrics used in this study. This tool provides a framework for researchers to select the best method for the normalization of their gene expression data based on the evaluation of different methods in the principle proposed in this study.

## Results

### Basic Concepts

**FIGURE 1:**
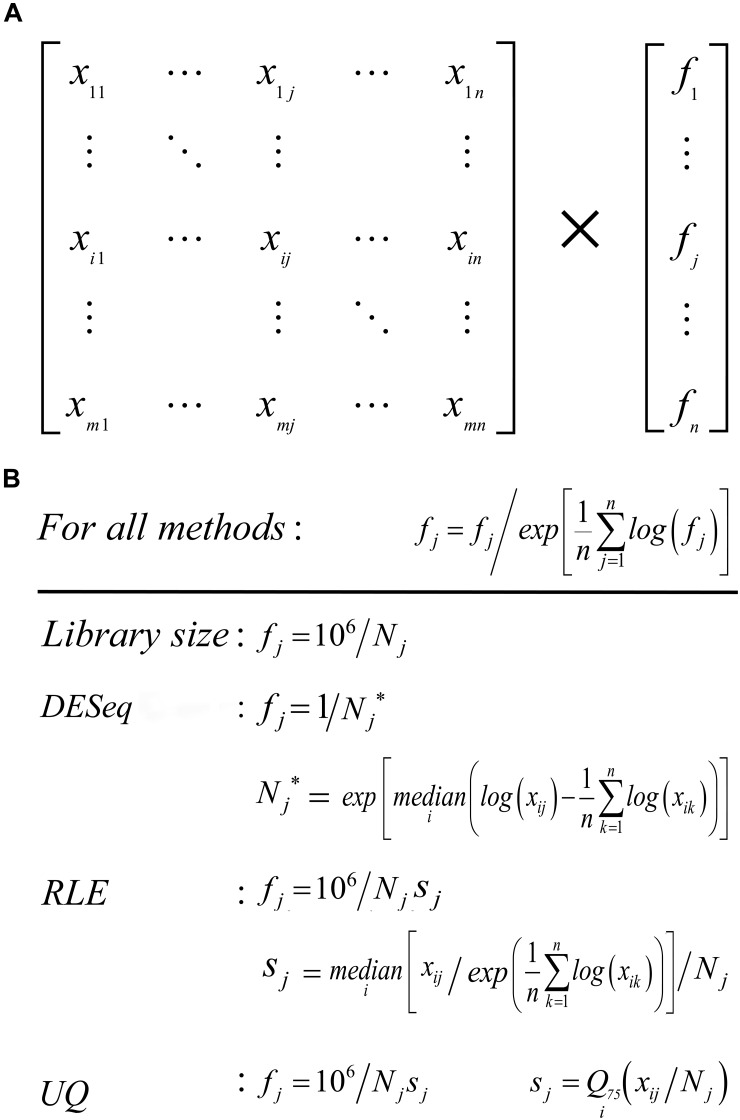
Basic concepts. **(A)** A raw gene expression matrix can be transformed into a normalized gene expression matrix by the multiplication of a global factor f_j_ to each column. Each column represents the expression levels of all genes from a sample and each row represents the expression levels of a gene across all samples. **(B)** As the library size methods, TN, TC, CR, or NR can be used to estimate a library size N_j_. The library size methods (TN, TC, CR, and NR) produce the global normalization factor f_j_ by the reciprocal of library size N_j_. HG7, ERCC, DESeq, TU, NCS, and ES produce a pseudo library size N_j_^∗^, which represents the relative amount of total RNA. RLE, UQ, and TMM produce a normalization factor s_j_ to normalize the library size N_j_ and the global normalization factor for data normalization should be 10^6^/N_j_s_j_. Q_75_ represents the third quartile Q_3_. For all methods, log represents the natural logarithm.

In total, 14 normalization methods have been evaluated in this study. They are Housekeeping Genes (HG7), External RNA Control Consortium (ERCC), Total Read Number (TN), Total Read Count (TC), Cellular RNA (CR), Nuclear RNA (NR), median of the ratios of observed counts (DESeq), Relative Log Expression (RLE), UQ, Trimmed Mean of M values (TMM), Total Ubiquitous (TU), Network Centrality Scaling (NCS), Evolution Strategy (ES), and SCnorm (see section “Materials and Methods”). Currently, most methods with a few exceptions (e.g., SCnorm) are used to normalize a raw gene expression matrix (n samples by m genes) by multiplying a global normalization factor to each of its columns, yielding a normalized gene expression matrix (Figure 1A). In different methods, the definitions of normalization factor, scaling factor and size factor are inconsistent and need to be explained here. Both the normalization factor defined in the package NormExpression and the scaling factor defined in a previous study (Glusman et al., 2013) are the global normalization factors (Figure 1A). As the library size methods, TN, TC, CR, or NR can be used to estimate a library size, which represents the amount of total RNA in a cDNA library from a sample. HG7, ERCC, DESeq, TU, NCS, and ES produce a pseudo library size (in Figure 1B), which represents the relative amount of total RNA. Library size is also named as size factor in the Bioconductor package DESeq (Anders and Huber, 2010). In general, HG7, ERCC, TN, TC, CR, NR, DESeq, TU, NCS, and ES produce the global normalization factor by the reciprocal of library size or pseudo library size. RLE, UQ, and TMM in the Bioconductor package edgeR (Robinson et al., 2010) produce normalization factors to normalize the library sizes and the global normalization factors for data normalization should be calculated by one million multiplying the reciprocal of normalized library sizes (Figure 1B). However, the normalization factors produced by RLE, UQ, and TMM have been wrongly used as the global normalization factors in previous studies (Li et al., 2015). The NormExpression package includes such modifications as below to integrate the above normalization methods. DESeq, RLE, UQ, and TMM have been modified to ignore zero values to be fit for the scRNA-seq data processing. As NR is the best among the library size methods (TN, TC, CR, and NR), RLE, UQ, and TMM use NR to estimate library sizes. As HG7 and ERCC produce pseudo library sizes (Figure 1B) as TN, TC, CR, and NR, their normalization factors are amplified by one million for a uniform representation (Figure 1B). The resulting normalization factors of all 14 methods except SCnorm need to be further normalized by their geometric mean values (Figure 1B). After further normalization, RLE is identical to DESeq and presented as DESeq (RLE) or DESeq^∗^ in this study. It has been confirmed that all the modifications do not change the evaluation results.

### Evaluation of 14 Normalization Methods

In the previous study, Glusman et al. (2013) had quantified the success of normalization methods by the number of uniform genes (see section “Materials and Methods”) and used the Coefficient of Variation (CV) cutoff 0.25 to determine the number of uniform genes for each method. This metric was designed based on the theory that the relative values among different normalization methods are quite stable, although the absolute number of uniform genes depend on the cutoff value. However, it is almost impossible to determine a CV cutoff for scRNA-seq data as CV in scRNA-seq data has a much larger dynamic range than in bulk RNA-seq data. Inspired by Area Under the receiver operating characteristic Curve (AUC) (Gao et al., 2009), we designed a new metric named Area Under normalized CV threshold Curve (AUCVC) to evaluate normalization methods. Using one scRNA-seq dataset scRNA663 and one bulk RNA-seq dataset bkRNA18 (see section “Materials and Methods”), we applied AUCVC and another metric mSCC (see section “Materials and Methods”) to evaluate 14 normalization methods and then we compared the evaluation results by mSCC with those by AUCVC to assess the consistency of datasets and the consistency of metrics.

**FIGURE 2:**
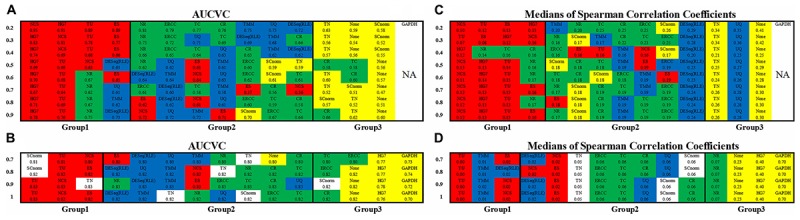
Consistency of metrics and consistency of datasets. The non-zero ratio cutoffs from 0.2 to 0.9 for scRNA663 and from 0.7 to 1 for bkRNA18 were used to produce AUCVCs and mSCCs. All the normalization methods were classified into three groups based on their AUCVC values sorted in descending order (from the best to the poorest) using one scRNA-seq dataset scRNA663 **(A)** and one bulk RNA-seq dataset bkRNA18 **(B)**. These methods were also classified into three groups based on their mSCC values sorted in ascending order (from the best to the poorest) using one scRNA-seq dataset scRNA663 **(C)** and one bulk RNA-seq dataset bkRNA18 **(D)**. GAPDH is not applicable to scRNA-seq data due to zero counts of GAPDH present in many samples. All the numbers are accurate to two decimal places, the marginal differences are reflected by their orders. The raw gene expression data (None) was also used to produce AUCVCs and mSCCs for comparison. After further normalization, RLE is identical to DESeq and presented as DESeq (RLE) or DESeq^∗^ in this study.

The non-zero ratio cutoffs (see section “Materials and Methods”) from 0.2 to 0.9 for scRNA663 and from 0.7 to 1 for bkRNA18 were used to produce AUCVCs of all methods (Figure 2A,B). Among 14 methods, TU, NCS, and ES are parameter-dependent approaches, which use the occurrence rate, upper and lower cutoffs as three parameters (see section “Materials and Methods”). For each non-zero ratio cutoff, TU used the maximum AUCVC to determine the optimal ones by testing all possible combinations of three parameters. In addition, the calculation only considered each combination of three parameters which produced more than 100 ubiquitous genes (see section “Materials and Methods”) for scRNA663 and more than 1,000 for bkRNA18. The occurrence rate cutoff was tested from 0.2 to 0.6 for scRNA663 at interval of 0.1 and set to 1 for bkRNA18. The lower cutoff was tested from 5 to 40% and the upper cutoff was tested from 60 to 95% at interval of 5%. For each non-zero ratio cutoff, NCS and ES used the occurrence rate, lower and upper cutoffs determined by TU, when TU achieved the maximum AUCVC.

The evaluation results using both scRNA663 and bkRNA18 showed consistency (the consistency of datasets) that all methods except HG7, TN and SCnorm were classified into three groups, based on their AUCVC values sorted in descending order (Figure 2A,B). The first group including TU, NCS and ES achieved the best performances. TU, NCS and ES, which had only been evaluated using bulk RNA-seq data in the previous study (Glusman et al., 2013), were evaluated by our new metric AUCVC as the best normalization methods using both scRNA-seq and bulk RNA-seq data. The second group including ERCC, TC, CR, NR, DESeq, RLE, UQ, and TMM achieved medial performances using both scRNA663 and bkRNA18. In the second group, ERCC, TC, CR, and NR outperformed DESeq, RLE, UQ, and TMM using scRNA663 but underperformed them using bkRNA18. The third group achieved the poorest performances, including TN, SCnorm and None (the raw gene expression data) for scRNA663 (Figure 2A), and HG7, GAPDH and None for bkRNA18 (Figure 2B). The evaluation results of HG7, TN, and SCnorm did not achieve the consistency using scRNA663 and bkRNA18. HG7 and GAPDH achieved the poorest performances using bkRNA18, suggesting that a predefined set of housekeeping genes may not be appropriate guides for the normalization of bulk RNA-seq data. However, it could be coincidental that HG7 was classified into the first group using scRNA663. TN underperformed the second group of methods using scRNA663 but outperformed it using bkRNA18. SCnorm was designed to improve the normalization of scRNA-seq data but it performed poorer using scRNA-seq data than bulk RNA-seq data. Particularly, SCnorm ranked the first in the best group by its AUCVC to normalize bkRNA18 when the non-zero ratio cutoffs were set to 0.7 or 0.8 (Figure 2B), but ranked the last in the poorest group to normalize scRNA663 when the non-zero ratio cutoffs were set to 0.2–0.4 (Figure 2A). SCnorm claimed that it is not designed to process datasets containing more than 80% zero counts. However, scRNA663 was build using the Smart-seq2 scRNA-seq protocol, which contained the least zero counts among current scRNA-seq protocols.

**FIGURE 3:**
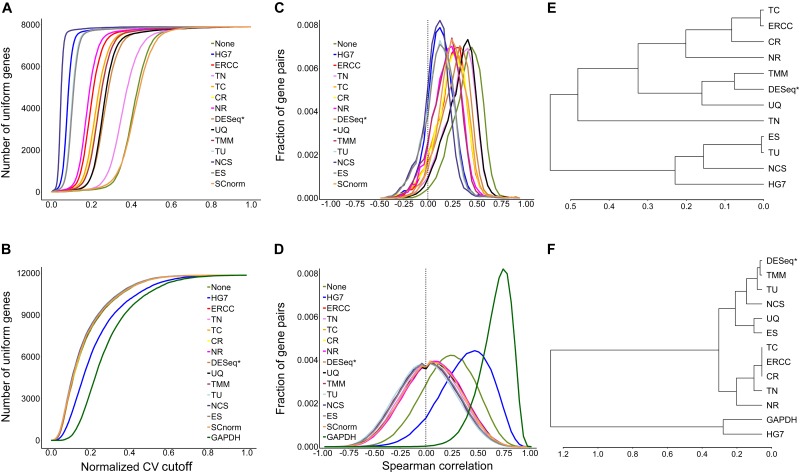
Visualization of evaluation results. A normalization method with a higher AUCVC produced a lower median of Spearman’s rank Correlation Coefficient (mSCC) between the normalized expression profiles of ubiquitous gene pairs using both scRNA-seq **(A,C)** and bulk RNA-seq data **(B,D)**. Using 1-SCCs as distances, hierarchical clustering of 13 normalization factors showed equivalent classification into the same groups **(E,F)** as those by AUCVC and by mSCC (Figure 2). SCnorm was not applicable to be used to calculate SCCs, as it produced a factor matrix rather than a factor vector as 13 other methods. GAPDH is not applicable to scRNA-seq data due to zero counts of GAPDH present in many samples. The raw gene expression data (None) was also used to produce AUCVCs and mSCCs for comparison. After further normalization, RLE is identical to DESeq and presented as DESeq (RLE) or DESeq^∗^ in this study.

The evaluation results (Figure 2C,D) by mSCC were consistent with those by AUCVC (the consistency of metrics). This proved that a successful normalization method simultaneously maximizes the number of uniform genes and minimizes the correlation between the expression profiles of gene pairs. We selected the best evaluation results using scRNA-seq data (the none-zero ratio cutoff = 0.2) and bulk RNA-seq data (the none-zero ratio cutoff = 1) in Figure 2 for visualization using NormExpression (Figure 3). Then, we calculated the Spearman’s rank Correlation Coefficients (SCCs) between all the normalization factors except that using SCnorm. Using 1-SCCs as distances, hierarchical clustering of 13 normalization factors showed equivalent classification into the same groups (Figure 3E,F) as those by AUCVC and by mSCC (Figure 2). From Figure 3, it can be seen that while all normalization methods except HG7, GAPDH and None performed without much differences using bulk RNA-seq data (Figure 3B,D), they had significant differences in the performances using scRNA-seq data (Figure 3A,C). These differences suggest that although scRNA-seq provides a higher resolution of cellular differences, it is more challenging to select the best method for the normalization of scRNA-seq data. In addition, these differences provided an explanation as to why scRNA-seq data and bulk RNA-seq data from the same samples resulted in different results in many previous studies. From Figure 3, it also can be seen that the best mSCC (TU-normalized) using scRNA-seq data still had a certain distance from 0 (Figure 3C), while the best mSCC (TU-normalized) using bulk RNA-seq data was close to 0 (Figure 3D). Therefore, further studies need to be conducted to investigate whether we can obtain the best mSCC using scRNA-seq data close to 0 as that using bulk RNA-seq data. If we cannot, what is the reason? And is it the nature of scRNA-seq data that result in this bias from 0?

To further test our principle, we searched other performance metrics in the published papers. The Bioconductor package scone (Cole et al., 2018) provides eight metrics to evaluate the normalization methods using scRNA-seq data. Among eight metrics, three are based on clustering properties and three other metrics are associated with control genes or QC metrics. Only two metrics based on global distributional properties can be used as general metrics. These two metrics are named as mean squared median relative log-expression (RLE_MED) and variance of inter-quartile range (IQR) of RLE (RLE_IQR). The evaluation results (Supplementary File 1) of three groups (particularly TU and ES) by RLE_MED were consistent with those by mSCC and by AUCVC using both scRNA-seq and bulk RNA-seq data. However, the evaluation results (Supplementary File 1) by RLE_IQR were not consistent with those by mSCC and by AUCVC. This suggests that mSCC, AUCVC, RLE_MED can be used together for method evaluation to test the consistency of metrics.

### Implementation and Availability

The raw gene expression data, normalization methods (except NCS, ES and SCnorm) and evaluation metrics (AUCVC and mSCC) have been included in the R package NormExpression. The data process in this study is provided in detail (Supplementary File 1). All the methods except NCS and ES have been implemented in R programs for their running on R platforms of any version. DESeq uses an R program from the Bioconductor package DESeq (Anders and Huber, 2010), which has been modified to process scRNA-seq data. RLE, UQ and TMM use R programs from the Bioconductor package edgeR (Robinson et al., 2010), which have been modified to process scRNA-seq data. NCS and ES had been implemented in Perl programs with multiple dependencies on Perl modules (Glusman et al., 2013), which have been modified into stand-alone programs for Linux systems (Supplementary File 2). SCnorm uses the Bioconductor package SCnorm (Bacher et al., 2017).

NormExpression can be used in three modes: normalization without evaluation, normalization with simple evaluation or normalization with complete evaluation. In the first mode, TU is recommended for the normalization of gene expression data, as it has been already ranked as the best method for both scRNA-seq and bulk RNA-seq data. In the second mode, AUCVC is used to select the best method from 10 normalization methods, which are HG7, ERCC (if available), TN, TC, CR, NR, DESeq (RLE), UQ, and TMM. TN, NCS, ES, and SCnorm are not used in the second mode, as the evaluation results of TN and SCnorm cannot achieve consistency, and NCS and ES have similar performances to TU but are much more time consuming. In the third mode, AUCVC and mSCC are used to select the best method from TU and at least 10 normalization methods. The normalization with simple evaluation determines the best method based on AUCVC values, while the normalization with complete evaluation determines the best method in the principle of the consistency of metrics and the consistency of datasets. As a result of a complete evaluation, the tables of AUCVC and mSCC (Figure 2) are required for the method selection.

## Materials and Methods

### Datasets

In a previous study (SRA: SRP113436), 831 single-cell samples and 18 bulk samples had been sequenced using the Smart-seq2 scRNA-seq protocol. In this study, we built a scRNA-seq dataset including 663 single cells from colon tumor tissues and 10 single cells from distal tissues (>10 cm) as control. The data of 166 single-cell samples were removed, as each of them contained NR less than 100,000 reads. The data of two single-cell samples were removed, as each of them contained simulated ERCC RNA less than 0 reads. We also built a bulk RNA-seq dataset including nine samples from colon tumor tissues and nine samples from distal tissues. The cleaning and quality control of both scRNA-seq and bulk RNA-seq data were performed using the pipeline Fastq_clean (Zhang et al., 2014) that was optimized to clean the raw reads from Illumina platforms. Using the software STAR (Dobin et al., 2013) v2.5.2b, we aligned all the cleaned scRNA-seq and bulk RNA-seq reads to the human genome GRCh38/hg38 and quantified the expression levels of 57,992 annotated genes (57,955 nuclear and 37 mitochondrial). Mitochondrial RNAs should have been, but were not discarded to test the robustness of normalization methods. Non-polyA RNAs and small RNAs (<200 bp) were not discarded either, although the Smart-seq2 protocol theoretically had only captured polyA RNAs. In addition, the expression levels of 92 ERCC RNAs and the long non-coding RNA (lncRNA) MDL1 in the human mitochondrial genome (Gao et al., 2017) were also quantified. ERCC RNA had been spiked into 208 single-cell samples before library construction; the expression levels of 92 ERCC RNAs in other 455 single-cell samples and 18 bulk samples were simulated by linear regression. Finally, the two datasets were named scRNA663 (58085 × 663) and bkRNA18 (58085 × 18), and used as raw gene expression data in this study. As these two datasets were obtained by sequencing the libraries using the same protocol and samples from the same group of patients, they had great values to be used to evaluate normalization methods and assess the consistency of datasets. Researchers can select the best method for the normalization of their gene expression data or evaluate different methods using the data of 57,955 nuclear genes.

### Normalization Methods

The library size methods (TN, TC, CR, and NR) use the gene expression level summed over total genes in a sample as the library size to calculate the normalization factor. HG7, ERCC and TU use the gene expression level summed over these pre-selected genes in a sample as the pseudo library size (see section “Results”). NR only counts reads which can be aligned to nuclear genomes, while CR counts reads which can be aligned to both nuclear and mitochondrial genomes. TC counts reads which can be aligned to 92 ERCC RNAs, nuclear and mitochondrial genomes (TC = CR + ERCC). TN uses the number of all reads which can be aligned to 92 ERCC RNAs, nuclear and mitochondrial genomes. The pre-selected genes used by HG7, ERCC and TU are seven housekeeping genes, 92 ERCC RNAs and the ubiquitous genes (described below), respectively. Seven genes (UBC, HMBS, TBP, GAPDH, HPRT1, RPL13A, and ACTB) in HG7 had been used to achieve the best evaluation result among those using all possible combinations of tested housekeeping genes in the previous study by Glusman et al. (2013). ERCC RNA is a set of commonly used spike-in RNA consisting of 92 polyadenylated transcripts with short 3′ polyA tails but without 5′ caps (Jiang et al., 2011). A single housekeeping gene GAPDH was used for comparison in the evaluation of normalization methods using bulk RNA-seq data, but it was not applicable to scRNA-seq data due to zero counts of GAPDH present in many samples. The raw gene expression data (None) was also used to produce AUCVCs and mSCCs for comparison.

### Uniform Genes and Ubiquitous Genes

A gene is defined as uniform when the Coefficient of Variation (CV, Formula 1) of its expression values across all samples is not more than a cutoff (Glusman et al., 2013). To determine the number of uniform genes using scRNA-seq data containing a high frequency of zeros, NormExpression only considers genes with non-zero ratios not less than a cutoff. The non-zero ratio of one gene should be calculated as the number of its all non-zero expression values divided by the number of total samples.

Ubiquitous genes are defined as the intersection of a trimmed sets of all samples (Glusman et al., 2013). This trimmed set of genes are selected for each sample by (1) excluding genes with zero values, (2) sorting the non-zero genes by their expression levels in that sample, and (3) removing the upper and lower ends of the sample-specific expression distribution. Glusman et al. (2013) determined the optimal parameters by testing all possible combinations of lower and upper cutoffs at interval of 5% to maximize the number of resulting uniform genes using one bulk RNA-seq dataset. The size of a scRNA-seq dataset is usually very large, which could result in a very small or even empty set of ubiquitous genes, as the number of ubiquitous genes depends on the sizes of datasets. To identify the ubiquitous genes using scRNA-seq data, we defined a parameter named occurrence rate, governing the minimal fraction of trimmed sets in which a gene must appear to be considered ubiquitous. In NormExpression, TU includes three parts. The first part determines the optimal parameters by testing all possible combinations of occurrence rate, lower and upper cutoffs to maximize AUCVC (described below) instead of the number of resulting uniform genes. The second part uses the optimal occurrence rate, upper and lower cutoffs to obtain the ubiquitous genes. The third part uses the ubiquitous genes to calculate the TU normalization factor. NormExpression only use the raw gene expression data to obtain the ubiquitous genes, which are used to calculate the TU normalization factor and to evaluate all methods. In addition, the same ubiquitous genes are used by NCS and ES to obtain the NCS and ES normalization factors, respectively. These ubiquitous genes are also used by TU, NCS and ES to produce their mSCCs for method evaluation.

### AUCVC and mSCC

In the previous study, Glusman et al. (2013) designed two novel and mutually independent metrics, which were the number of uniform genes and Spearman’s rank Correlation Coefficients (SCCs) between expression profiles of gene pairs. The basic theory underlying these two evaluation metrics is that a successful normalization method simultaneously maximizes the number of uniform genes and minimizes the correlation between the expression profiles of gene pairs. In this study, we designed a new metric AUCVC instead of the number of uniform genes and used the median of Spearman’s rank Correlation Coefficients between the normalized expression profiles of ubiquitous gene pairs (mSCC) instead of observation of SCC distributions for method evaluation. On default settings, NormExpression randomly selected 1,000,000 ubiquitous gene pairs to calculate the mSCCs for method evaluation (Figure 3C,D).

AUCVC (Figure 3A,B) is created by plotting the number of uniform genes (y-axis) at each normalized CV (Formula 2) cutoff (*x*-axis). As a high or a low normalized CV cutoff produces more false or less true uniform genes, it is reasonable to consider the overall performance of each method at various cutoff settings instead of that at one specific cutoff setting. In Formula 1 and 2, symbols have the same meanings as those in Figure 1 and n^∗^ does not count zero elements in each sample.

(1) $${\mathrm{CV}}_{i}=\left(\sqrt{\frac{1}{n-1}\sum_{j=1}^{n}{\left(x_{\mathrm{ij}}-{\bar{x}}_{i}\right)}^{2}}\right)/{\bar{x}}_{i},\;{\bar{x}}_{i}=\frac{1}{n}\sum_{j=1}^{n}x_{\mathrm{ij}}$$

(2) $$\begin{array}{l} {\mathrm{Normalized CV}}_{i}=\left\{{\mathrm{CV}}_{i}-\underset{i}{\min}({\mathrm{CV}}_{i})\right\}/\left\{\underset{i}{\max}({\mathrm{CV}}_{i})-\underset{i}{\min}({\mathrm{CV}}_{i})\right\}\\ {\mathrm{CV}}_{i}=\left(\sqrt{\frac{1}{n*-1}\sum_{j=1}^{n*}{\left(\log_{2}(x_{\mathrm{ij}})-{\bar{x}}_{i}\right)}^{2}}\right)/{\bar{x}}_{i},\;{\bar{x}}_{i}=\frac{1}{n*}\sum_{j=1}^{n*}\log_{2}(x_{\mathrm{ij}}),\\ \;x_{\mathrm{ij}}>0 \end{array}$$

## Author Contributions

SG conceived this project. SG and JR supervised this project. ZW, WL, and XJ performed the programming. ZW and HJ analyzed the data. HW prepared all the figures and Supplementary Files. SG drafted the main manuscript. GG, MR, and LL revised the manuscript. All authors read and approved the manuscript.

## Conflict of Interest Statement

The authors declare that the research was conducted in the absence of any commercial or financial relationships that could be construed as a potential conflict of interest.

## Supplementary Material

FILE S1 How to use NormExpression.

FILE S2 Perl scripts to run NCS and ES.
