# Supplementary material for: NormExpression: An R Package to Normalize Gene Expression Data Using Evaluated Methods
Source: Front Genet. 2019 Apr 30;10:400. doi: 10.3389/fgene.2019.00400 (PMC6503164; doi:10.3389/fgene.2019.00400)

NormExpression [1](#__RefHeading___Toc505802382)

1 Normalization with simple evaluation [2](#__RefHeading___Toc505802383)

1.A R package installation and data preparation [2](#__RefHeading___Toc505802384)

1.B How to produce scRNA663_factors (single-cell data) [3](#__RefHeading___Toc505802385)

1.C Normalization without evaluation (single-cell data) [4](#__RefHeading___Toc505802386)

1.D Normalization without evaluation (bulk data) [4](#__RefHeading___Toc505802387)

1.E Using AUCVC for simply evaluation (single-cell data) [5](#__RefHeading___Toc505802388)

1.F Using AUCVC for simply evaluation (bulk data) [5](#__RefHeading___Toc505802389)

2 Normalization with complete evaluation [6](#__RefHeading___Toc505802390)

2.A Using AUCVC for complete evaluation (single-cell data) [6](#__RefHeading___Toc505802391)

2.B Using AUCVC for complete evaluation (bulk data) [7](#__RefHeading___Toc505802392)

2.C Using mSCC for complete evaluation (single-cell data) [8](#__RefHeading___Toc505802393)

2.D Using mSCC for complete evaluation (bulk data) [9](#__RefHeading___Toc505802394)

3 Visualization of evaluation results [10](#__RefHeading___Toc505802395)

3.A CV threshold Curve (single-cell data) [10](#__RefHeading___Toc505802396)

3.B CV threshold Curve (bulk data) [11](#__RefHeading___Toc505802397)

3.C Distribution of SCCs between genes (single-cell data) [12](#__RefHeading___Toc505802398)

3.D Distribution of SCCs between genes (bulk data) [13](#__RefHeading___Toc505802399)

3.E Hierarchical clustering of normalization factors (single-cell data) [14](#__RefHeading___Toc505802400)

3.F Hierarchical clustering of normalization factors (bulk data) [15](#__RefHeading___Toc505802401)

4 How to evaluate many users' methods [16](#__RefHeading___Toc505802402)

4.A For methods with normalization factors (single-cell data) [16](#__RefHeading___Toc505802403)

4.B For a general usage (single-cell data) [17](#__RefHeading___Toc505802404)

5 Package versions and FAQ [17](#__RefHeading___Toc505802405)

# NormExpression

NormExpression provides a framework and a fast and simple way for researchers to evaluate methods (particularly some data-driven methods or their own methods) and then select a best one for data normalization in the gene expression analysis, based on the theory that a successful normalization method simultaneously maximizes the number of uniform genes and minimizes the correlation between the expression profiles of gene pairs. To evaluate normalization methods, NormExpression uses two metrics, which are AUCVC and mSCC [1].

NormExpression can be used in three ways: normalization without evaluation, normalization with simple evaluation or normalization with complete evaluation. (1) Normalization without evaluation is suggested to use TU to process users' data, since it has been already ranked as the best method for both scRNA-seq (**1.C**) and bulk RNA-seq data (**1.D**) [1]. (3) Normalization with simple evaluation by AUCVC is suggested to select the best method from 10 normalization methods using users' single-cell data (**1.E**) or bulk data (**1.F**), which are HG7, ERCC (if available), TN, TC, CR, NR, DESeq (RLE), UQ and TMM. (3) Normalization with complete evaluation by AUCVC and mSCC is suggested to select the best method from at least 10 normalization methods plus TU, using users' single-cell data (**2.AC**) or bulk data (**2.BD**). Normalization with simple evaluation determines the best method based on its AUCVC value, while normalization with complete evaluation need consider the consistency of evaluation results using two metrics (**the consistency of metrics**). We suggest to use (3) as the best choice, although **it is time-consuming**. If users chose to use (1), we suggest them to conduct (2) and compare the results with our previous results [1] to validate (1). This consistency can also be used to analyze the quality of gene expression data.

**Notice:** (1) Users can run all the R coding as below by "copy-paste"; (2) To use NormExpression, please cite this paper.

[1] Zhenfeng Wu, Weixiang Liu, Xiufeng Jin, Deshui Yu, Hua Wang, Gustavo Glusman, Max Robinson, Lin Liu, Jishou Ruan, Shan Gao (2018) NormExpression: an R package to normalize gene expression data using evaluated methods. bioRxiv. https://doi.org/10.1101/251140

Two datasets scRNA663 (single-cell data) and bkRNA18 (bulk data) can be used to evaluate users' methods. These two datasets were designed to use the same library-construction and sequencing protocol to validate the consistency of evaluations by two types of data (**the consistency of datasets**). Users should select the best one for their data normalization based on the evaluated methods using their own data. The gene expression data of scRNA663 will be released after the relevant paper is published. Before the release day, users can request the data by emailing Shan Gao (gao_shan@mail.nankai.edu.cn) but need provide their names and affiliated institutions.

## 1 Normalization with simple evaluation

### 1.A R package installation and data preparation

| # Set the working directory  setwd("d:/working_dir");  # Install R package  install.packages("NormExpression");  install.packages("ggplot2");  install.packages("dendextend");  # Load libraries  library(NormExpression);  library(ggplot2);  library(dendextend);  # Read all data  # scRNA663 will be released after the relevant paper is published.  data(scRNA663);  # Readers can request scRNA663.txt and read it by  scRNA663 <- read.table(file='scRNA663.txt', header=TRUE, row.names=1);  # scRNA663_factors contains all the pre-calculated normalization factors using scRNA663  data(scRNA663_factors);  # bkRNA18 is bulk data  data(bkRNA18);  # bkRNA18_factors contains all the pre-calculated normalization factors using bkRNA18  data(bkRNA18_factors);  # Use SCnorm to produce the normalized gene expression matrix (single-cell data)  source("https://bioconductor.org/biocLite.R);  biocLite("SCnorm");  library(SCnorm);  Conditions = rep(c(1), each= 663);  pdf("scRNA663_count-depth_norm.pdf", height=7.5, width=10.5);  DataNorm <- SCnorm(Data = scRNA663, Conditions = Conditions, FilterExpression = 4, PrintProgressPlots = TRUE, reportSF = TRUE, NCores=1);  dev.off();  NormalizedData <- results(DataNorm);  scRNA663.SCnorm <- round(NormalizedData, 2);  # Use SCnorm to produce the normalized gene expression matrix (bulk data)  Conditions = rep(c(1), each= 18);  pdf("bkRNA18_count-depth_norm.pdf", height=7.5, width=10.5);  DataNorm <- SCnorm(Data = bkRNA18, Conditions = Conditions, FilterExpression = 5, PrintProgressPlots = TRUE, reportSF = TRUE, NCores=1);  dev.off();  NormalizedData <- results(DataNorm);  bkRNA18.SCnorm <- round(NormalizedData, 2); |
| --- |

Results:


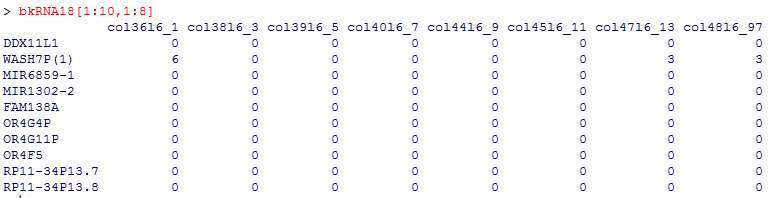


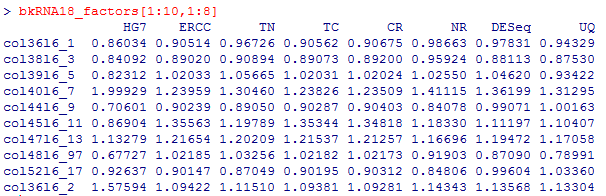


### 1.B How to produce scRNA663_factors (single-cell data)

| # bkRNA18_factors can be produced in the same way  # TU, NCS and ES are parameter dependent methods  # So, scRNA663_factors contain TU, NCS and ES normalization factors produced using the optimal parameters  # For HG7, ERCC, TC, CR and NR  housekeeping.list <- read.table(file='housekeeping.txt', header = FALSE);  hk_name <- as.matrix(housekeeping.list)[,1];  HG7.size <- colSums(scRNA663[hk_name,]);  HG7_factors <- getFactors(data=scRNA663, lib.size=HG7.size, method="sizefactor");  # For DESeq(RLE), UQ and TMM  DESeq_factors <- getFactors(data= scRNA663, method="DESeq");  ……  scRNA663_factors <- cbind(HG7_factors, DESeq_factors……);  colnames(scRNA663_factors)=c("HG7", "ERCC", "TN", "TC", "CR", "NR", "DESeq", "UQ", "TMM", "TU", "NCS", "ES"); |
| --- |

### 1.C Normalization without evaluation (single-cell data)

| # Grid of non-zero ratios to produce AUCVCs for TU and **it is time-consuming**  scRNA663.AUCVCs1 <- gridAUCVC(data= scRNA663, dataType="sc", TU= 1, nonzeroRatios= c(0.2,0.3,0.4,0.5,0.6,0.7,0.8,0.9));  # Look at the maximum AUCVC for TU  scRNA663.AUCVCs1;  # Find the parameters used by TU when it achieved the maximum AUCVC in bestPara.txt  bestPara <- read.table(file='bestPara.txt', header=TRUE);  bestPara;  # Using the parameters to produce the TU normalization factor  tu <- getFactors(data = scRNA663, method = "TU", pre_ratio=0.5, lower_trim=0.05, upper_trim=0.65);  # Produce the TU-normalized gene expression matrix  TU_sc.matrix <- getNormMatrix(data = scRNA663, norm.factors = tu); |
| --- |

### 1.D Normalization without evaluation (bulk data)

| # Grid of non-zero ratios to produce AUCVCs for TU and **it is time-consuming**  # nonzeroRatios can be set to 1 for bulk data to reduce computing time  bkRNA18.AUCVCs1 <- gridAUCVC(data= bkRNA18, dataType="bk", TU= 1, nonzeroRatios= c(0.7,0.8,0.9,1));  # Look at the maximum AUCVC for TU  bkRNA18.AUCVCs1;  # Find the parameters used by TU when it achieved the maximum AUCVC in bestPara.txt bestPara <- read.table(file='bestPara.txt', header=TRUE);  bestPara;  # Using the parameters to produce the TU normalization factor  tu <- getFactors(data = bkRNA18, method = "TU", pre_ratio=1, lower_trim=0.2, upper_trim=0.6);  # Produce the TU-normalized gene expression matrix  TU_bk.matrix <- getNormMatrix(data = bkRNA18, norm.factors = tu); |
| --- |

Results:

| 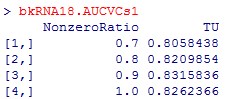 | 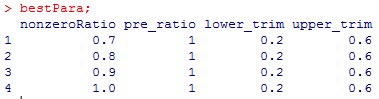 |
| --- | --- |


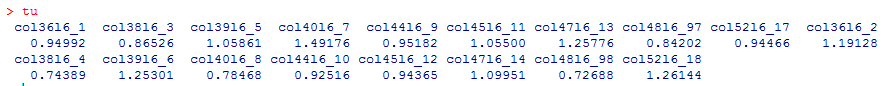


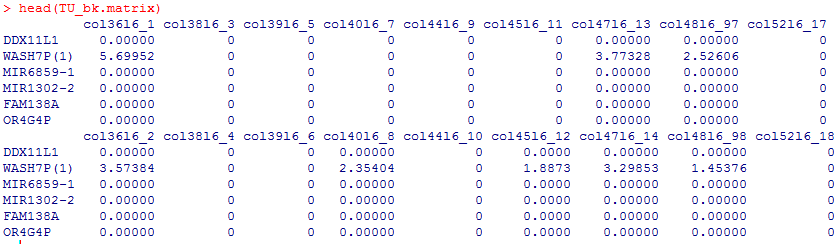


### 1.E Using AUCVC for simply evaluation (single-cell data)

| # Grid of nonzero ratios to produce AUCVCs for 10 methods  # Since RLE is identical to DESeq, only normalization factors of 9 methods are required  # scRNA663_factors contains these 9 normalization factors  # **TN can use one of user's normalization factors**  scRNA663.AUCVCs <- gridAUCVC(data= scRNA663, dataType="sc", HG7= scRNA663_factors$HG7, ERCC= scRNA663_factors$ERCC, TN= scRNA663_factors$TN, TC= scRNA663_factors$TC, CR= scRNA663_factors$CR, NR= scRNA663_factors$NR, DESeq= scRNA663_factors$DESeq, UQ= scRNA663_factors$UQ, TMM= scRNA663_factors$TMM, nonzeroRatios= c(0.2,0.3,0.4,0.5,0.6,0.7,0.8,0.9)); |
| --- |

Results(**2A [1]**): This need be compared with the AUCVCs of TU in **1.C**


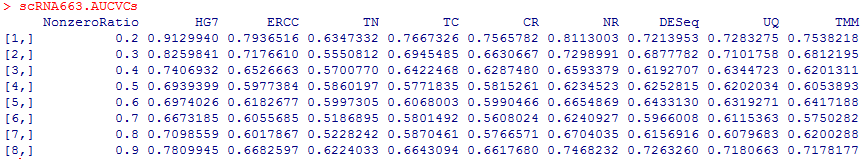


### 1.F Using AUCVC for simply evaluation (bulk data)

| # Grid of nonzero ratios to produce AUCVCs for 10 methods  # Since RLE is identical to DESeq, only normalization factors of 9 methods are required  # scRNA18_factors contains these 9 normalization factors  # **TN can use one of user's normalization factors**  bkRNA18.AUCVCs <- gridAUCVC(data= bkRNA18, dataType="bk", HG7=bkRNA18_factors$HG7, ERCC=bkRNA18_factors$ERCC, TN=bkRNA18_factors$TN, TC=bkRNA18_factors$TC, CR=bkRNA18_factors$CR, NR=bkRNA18_factors$NR, DESeq=bkRNA18_factors$DESeq, UQ=bkRNA18_factors$UQ, TMM=bkRNA18_factors$TMM, GAPDH=bkRNA18_factors$GAPDH, nonzeroRatios= c(0.7,0.8,0.9,1)); |
| --- |

Results(**2B[1]**): This need be compared with the AUCVCs of TU in **1.D**


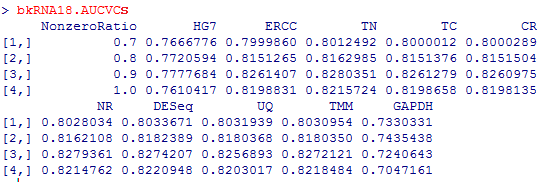


## 2 Normalization with complete evaluation

### 2.A Using AUCVC for complete evaluation (single-cell data)

| # Compared to **1.E**, the TU method need be added for comparison (**more time-consuming**)  # One more parameter need be set (TU=1) for the function gridAUCVC  # **TN can use one of user's normalization factors**  scRNA663.AUCVCs1 <- gridAUCVC(data= scRNA663, dataType="sc", HG7= scRNA663_factors$HG7, ERCC= scRNA663_factors$ERCC, TN= scRNA663_factors$TN, TC= scRNA663_factors$TC, CR= scRNA663_factors$CR, NR= scRNA663_factors$NR, DESeq= scRNA663_factors$DESeq, UQ= scRNA663_factors$UQ, TMM= scRNA663_factors$TMM, TU= 1, nonzeroRatios= c(0.2,0.3,0.4,0.5,0.6,0.7,0.8,0.9));  # Look at the maximum AUCVC for TU  scRNA663.AUCVCs1;  # Find the parameters used by TU when it achieved the maximum AUCVC in bestPara.txt  bestPara <- read.table(file='bestPara.txt', header=TRUE);  bestPara;  # Using the parameters to produce the TU normalization factor  tu <- getFactors(data = scRNA663, method = "TU", pre_ratio=0.5, lower_trim=0.05, upper_trim=0.65); |
| --- |

**This section is optional**

| # NCS, ES and SCnorm are not integrated into NormExpression  # Produce the NCS-normalized gene expression matrix (Nonzero ratio=0.2)  # (fraction, lower_trim_inclusive, upper_trim_inclusive) is from bestPara  # For nonzero ratios from 0.3 to 0.9, users need iteratively produce a new matrix  ./normalize.pl --infile scRNA663.txt --outfile NCS --method net --verbose 1 --fraction 0.5 --lower_trim_inclusive 5 --upper_trim_inclusive 65 --logmaxmincutoff 10 --limit_genes 0 --is_bulk 0 > NCS.log 2>&1 &  # Produce the ES-normalized gene expression matrix (Nonzero ratio=0.2)  # (fraction, lower_trim, upper_trim) is from bestPara  ./normalize.pl --infile scRNA663.txt --outfile ES --solution_file solutions_sc.tab --method evolution_strategy --verbose 1 --fraction 0.5 --lower_trim 5 --upper_trim 65 --CoV_cutoff 0.8 --all_genes 0 --time_to_spend 800000 --populationsize 11 --roundswithoutimprovement 10 --is_bulk 0 > ES.log 2>&1 &  # Produce AUCVCs for NCS, ES and SCnorm (Nonzero ratio=0.2)  NCS.matrix <- getNormMatrix(scRNA663, scRNA663_factors$NCS);  ES.matrix <- getNormMatrix(scRNA663, scRNA663_factors$ES);  scRNA663.AUCVCs2 <- gridAUCVC4Matrices(None= scRNA663, NCS=NCS.matrix, ES=ES.matrix, SCnorm= scRNA663.SCnorm, nonzeroRatios= 0.2);  # Produce AUCVCs for NCS, ES and SCnorm (Nonzero ratio=0.3 to 0.9)  # Iteratively add new_row to the result matrix scRNA663.AUCVCs2  scRNA663.AUCVCs2=rbind(scRNA663.AUCVCs2, new_row);  …….  # Combine two matrices  scRNA663.AUCVCs <- cbind(scRNA663.AUCVCs1, scRNA663.AUCVCs2); |
| --- |

### 2.B Using AUCVC for complete evaluation (bulk data)

| # Compared to **1.F**, the TU method need be added for comparison (**more time-consuming**)  # One more parameter need be set (TU=1) for the function gridAUCVC  # **TN can use one of user's normalization factors**  bkRNA18.AUCVCs1 <- gridAUCVC(data= bkRNA18, dataType="bk", HG7= bkRNA18_factors$HG7, ERCC= bkRNA18_factors$ERCC, TN=bkRNA18_factors$TN, TC=bkRNA18_factors$TC, CR=bkRNA18_factors$CR, NR=bkRNA18_factors$NR, DESeq=bkRNA18_factors$DESeq, UQ=bkRNA18_factors$UQ, TMM=bkRNA18_factors$TMM, TU= 1, GAPDH=bkRNA18_factors$GAPDH, nonzeroRatios= c(0.7, 0.8, 0.9, 1));  # Look at the maximum AUCVC for TU  bkRNA18.AUCVCs1;  # Find the parameters used by TU when it achieved the maximum AUCVC in bestPara.txt bestPara <- read.table(file='bestPara.txt', header=TRUE);  bestPara;  # Using the parameters to produce the TU normalization factor  tu <- getFactors(data = bkRNA18, method = "TU", pre_ratio=1, lower_trim=0.2, upper_trim=0.6); |
| --- |

**This section is optional**

| # NCS, ES and SCnorm are not integrated into NormExpression  # Produce the NCS-normalized gene expression matrix (Nonzero ratio=1)  # (fraction, lower_trim_inclusive, upper_trim_inclusive) is from bestPara  # For nonzero ratios from 0.7 to 0.9, users need iteratively produce a new matrix  ./normalize.pl --infile bkRNA18.txt --outfile NCS --method net --verbose 1 --fraction 1 --lower_trim_inclusive 20 --upper_trim_inclusive 60 --logmaxmincutoff 3 --limit_genes 0 --is_bulk 1 > NCS.log 2>&1 &  # Produce the ES-normalized gene expression matrix (Nonzero ratio=1)  # (fraction, lower_trim, upper_trim) is from bestPara  ./normalize.pl --infile bkRNA18.txt --outfile ES --solution_file solutions_bk.tab --method evolution_strategy --verbose 1 --fraction 1 --lower_trim 20 --upper_trim 60 --CoV_cutoff 0.25 --all_genes 0 --time_to_spend 800000 --populationsize 11 --roundswithoutimprovement 10 --is_bulk 1 > ES.log 2>&1 &  # Produce AUCVCs for NCS, ES and SCnorm (Nonzero ratio=1)  NCS.matrix <- getNormMatrix(bkRNA18, bkRNA18_factors$NCS);  ES.matrix <- getNormMatrix(bkRNA18, bkRNA18_factors$ES);  bkRNA18.AUCVCs2 <- gridAUCVC4Matrices(None= bkRNA18, NCS=NCS.matrix, ES=ES.matrix, SCnorm= bkRNA18.SCnorm, nonzeroRatios= 1);  # Produce AUCVCs for NCS, ES and SCnorm (Nonzero ratio=0.7 to 0.9)  # Iteratively add new_row to the result matrix scRNA663.AUCVCs2  bkRNA18.AUCVCs2=rbind(bkRNA18.AUCVCs2,new_row);  …….  # Combine two matrices  bkRNA18.AUCVCs <- cbind(bkRNA18.AUCVCs1, bkRNA18.AUCVCs2); |
| --- |

### 2.C Using mSCC for complete evaluation (single-cell data)

| # Produce mSCCs for 11 methods (Nonzero ratio=0.2)  # Since RLE is identical to DESeq, only normalization factors of 10 methods are required  # scRNA663_factors contains normalization factors of 9 methods  # The TU normalization factor is from tu (**2.A**)  # **TN can use one of user's normalization factors**  # (pre_ratio, lower_trim, upper_trim) is from bestPara (**2.A**)  # Users can produce mSCCs for 11 methods (Nonzero ratio=0.3-0.9) but not necessarily  scRNA663.cors1 <- gatherCors(data= scRNA663, cor_method="spearman", HG7= scRNA663_factors$HG7, ERCC= scRNA663_factors$ERCC, TN= scRNA663_factors$TN, TC= scRNA663_factors$TC, CR= scRNA663_factors$CR, NR= scRNA663_factors$NR, DESeq= scRNA663_factors$DESeq, UQ= scRNA663_factors$UQ, TMM= scRNA663_factors$TMM, TU= tu, pre_ratio=0.5, lower_trim=0.05, upper_trim=0.65, rounds=1000000); |
| --- |

**This section is optional**

| # Produce mSCCs for NCS, ES and SCnorm  # (pre_ratio, lower_trim, upper_trim) is from bestPara (**2.A**)  NCS.matrix <- getNormMatrix(scRNA663, scRNA663_factors$NCS);  ES.matrix <- getNormMatrix(scRNA663, scRNA663_factors$ES);  scRNA663.cors2 <- gatherCors4Matrices(None= scRNA663, NCS=NCS.matrix, ES=ES.matrix, SCnorm= scRNA663.SCnorm, raw_matrix= scRNA663, cor_method="spearman", pre_ratio=0.5, lower_trim=0.05, upper_trim=0.65, rounds=1000000);  # combine all mSCCs (**2C the first line[1]**)  scRNA663.cors <- rbind(scRNA663.cors1, scRNA663.cors2);  scRNA663.cor.medians <- getCorMedians(scRNA663.cors); |
| --- |

### 2.D Using mSCC for complete evaluation (bulk data)

| # Produce mSCCs for 11 methods (Nonzero ratio=1)  # Since RLE is identical to DESeq, only normalization factors of 10 methods are required  # bkRNA18_factors contains normalization factors of 9 methods  # The TU normalization factor is from tu (**2.B**)  # **TN can use one of user's normalization factors**  # (pre_ratio, lower_trim, upper_trim) is from bestPara (**2.B**)  # Users can produce mSCCs for 11 methods (Nonzero ratio=0.7-0.9) but not necessarily  bkRNA18.cors1 <- gatherCors(data= bkRNA18, cor_method="spearman", HG7= bkRNA18_factors$HG7, ERCC= bkRNA18_factors$ERCC, TN= bkRNA18_factors$TN, TC= bkRNA18_factors$TC, CR= bkRNA18_factors$CR, NR= bkRNA18_factors$NR, DESeq= bkRNA18_factors$DESeq, UQ= bkRNA18_factors$UQ, TMM= bkRNA18_factors$TMM, TU= tu, GAPDH= bkRNA18_factors$GAPDH, pre_ratio=1, lower_trim=0.2, upper_trim=0.6, rounds=1000000); |
| --- |

**This section is optional**

| # Produce mSCCs for NCS, ES and SCnorm  # (pre_ratio, lower_trim, upper_trim) is from bestPara (**2.A**)  NCS.matrix <- getNormMatrix(bkRNA18, bkRNA18_factors$NCS);  ES.matrix <- getNormMatrix(bkRNA18, bkRNA18_factors$ES);  bkRNA18.cors2 <- gatherCors4Matrices(None= bkRNA18, NCS=NCS.matrix, ES=ES.matrix, SCnorm= bkRNA18.SCnorm, raw_matrix=bkRNA18, cor_method="spearman", pre_ratio=1, lower_trim=0.2, upper_trim=0.6, rounds=1000000);  # combine all mSCCs (**2D the fourth line [1]**)  bkRNA18.cors <- rbind(bkRNA18.cors1, bkRNA18.cors2);  bkRNA18.cor.medians <- getCorMedians(bkRNA18.cors); |
| --- |

## 3 Visualization of evaluation results

### 3.A CV threshold Curve (single-cell data)

| # scRNA663_factors contains all the pre-calculated normalization factors (Nonzero ratio=0.2)  scRNA663.cv_uniform1 <- gatherCVs(data= scRNA663, nonzeroRatio= 0.2, HG7= scRNA663_factors$HG7, ERCC= scRNA663_factors$ERCC, TN= scRNA663_factors$TN, TC= scRNA663_factors$TC, CR= scRNA663_factors$CR, NR= scRNA663_factors$NR, DESeq= scRNA663_factors$DESeq, UQ= scRNA663_factors$UQ, TMM= scRNA663_factors$TMM, TU= scRNA663_factors$TU);  NCS.matrix <- getNormMatrix(scRNA663, scRNA663_factors$NCS);  ES.matrix <- getNormMatrix(scRNA663, scRNA663_factors$ES);  scRNA663.cv_uniform2 <- gatherCVs4Matrices(None= scRNA663, NCS=NCS.matrix, ES=ES.matrix, SCnorm= scRNA663.SCnorm, raw_matrix=scRNA663, nonzeroRatio=0.2);  scRNA663.cv_uniform <- rbind(scRNA663.cv_uniform1, scRNA663.cv_uniform2);  # plot  tiff(file = "scRNA663_cv.tif", res=300, compression = "lzw",width=(1200*4.17),height=(960*4.17));  plotCVs(scRNA663.cv_uniform, methods=c("None", "HG7", "ERCC", "TN", "TC", "CR", "NR", "DESeq", "UQ", "TMM", "TU", "NCS", "ES", "SCnorm"), legend.position=c(.85, .48));  dev.off(); |
| --- |

Results(**3A[1]**):


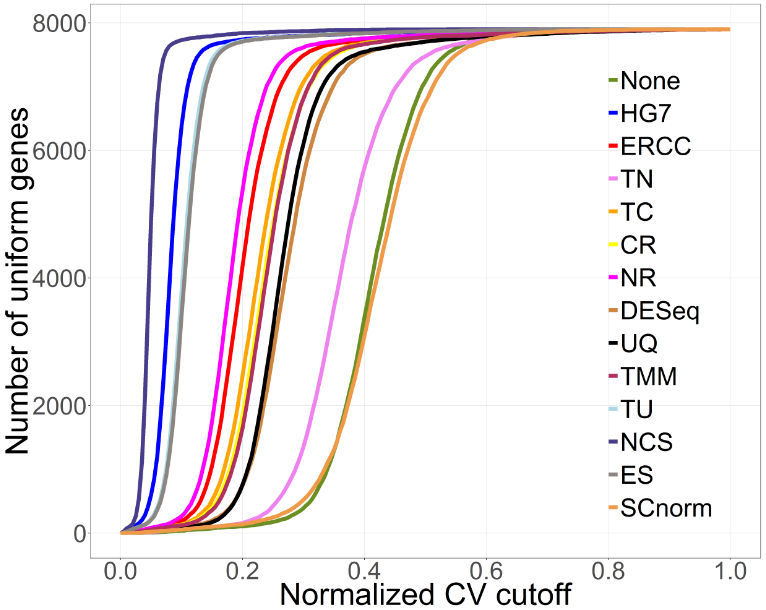


### 3.B CV threshold Curve (bulk data)

| # bkRNA18_factors contains all the pre-calculated normalization factors (Nonzero ratio=1)  bkRNA18.cv_uniform1 <- gatherCVs(data= bkRNA18, nonzeroRatio= 1, HG7= bkRNA18_factors$HG7, ERCC= bkRNA18_factors$ERCC, TN= bkRNA18_factors$TN, TC= bkRNA18_factors$TC, CR= bkRNA18_factors$CR, NR= bkRNA18_factors$NR, DESeq= bkRNA18_factors$DESeq, UQ= bkRNA18_factors$UQ, TMM= bkRNA18_factors$TMM, TU= bkRNA18_factors$TU, GAPDH = bkRNA18_factors$GAPDH);  NCS.matrix <- getNormMatrix(bkRNA18, bkRNA18_factors$NCS);  ES.matrix <- getNormMatrix(bkRNA18, bkRNA18_factors$ES);  bkRNA18.cv_uniform2 <- gatherCVs4Matrices(None= bkRNA18, NCS=NCS.matrix, ES=ES.matrix, SCnorm= bkRNA18.SCnorm, raw_matrix =bkRNA18, nonzeroRatio=1);  bkRNA18.cv_uniform <- rbind(bkRNA18.cv_uniform1, bkRNA18.cv_uniform2);  # plot  tiff(file = "bkRNA18_cv.tif", res=300, compression = "lzw",width=(1200*4.17),height=(960*4.17));  plotCVs(bkRNA18.cv_uniform, methods=c("None", "HG7", "ERCC", "TN", "TC", "CR", "NR", "DESeq", "UQ", "TMM", "TU", "NCS", "ES", "SCnorm", "GAPDH"), legend.position=c(.85, .48));  dev.off(); |
| --- |

Results(**3B[1]**):


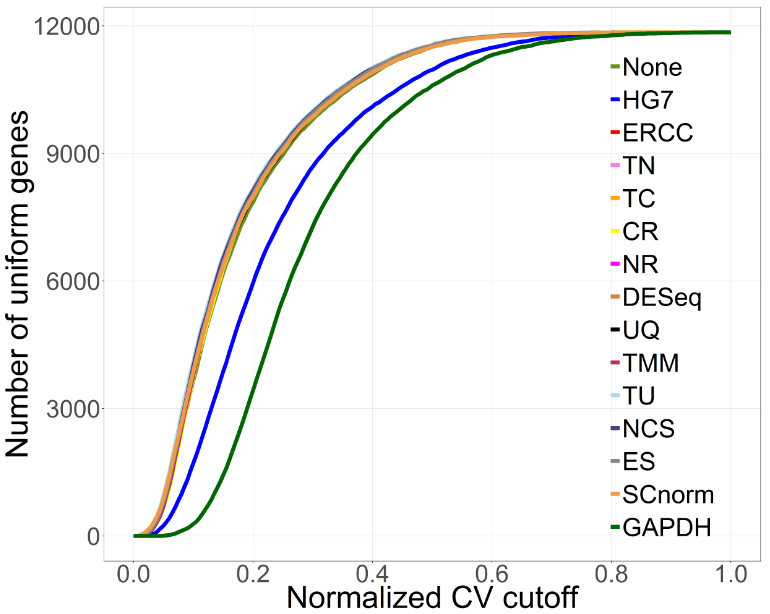


### 3.C Distribution of SCCs between genes (single-cell data)

| # scRNA663.cors is from **2.C** (Nonzero ratio=0.2)  tiff(file =" scRNA663_sp.tif", res=300, compression = "lzw",width=(1200*4.17),height=(960*4.17));  plotCors(scRNA663.cors, methods=c("None", "HG7", "ERCC", "TN", "TC", "CR", "NR", "DESeq", "UQ", "TMM", "TU", "NCS", "ES", "SCnorm"), legend.position=c(.15, .56))  dev.off(); |
| --- |

Results(**3C[1]**):


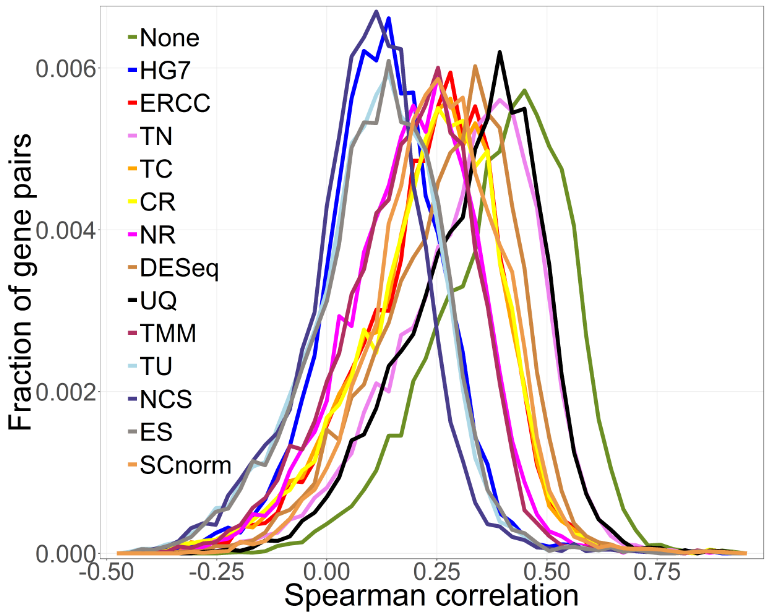


### 3.D Distribution of SCCs between genes (bulk data)

| # bkRNA18.cors is from **2.D** (Nonzero ratio=1)  tiff(file = "bkRNA18_sp.tif", res=300, compression = "lzw",width=(1200*4.17),height=(960*4.17));  plotCors(bkRNA18.cors, methods=c("None", "HG7", "ERCC", "TN", "TC", "CR", "NR", "DESeq", "UQ", "TMM", "TU", "NCS", "ES", "SCnorm", "GAPDH"), legend.position=c(.15, .56));  dev.off(); |
| --- |

Results(**3D[1]**):


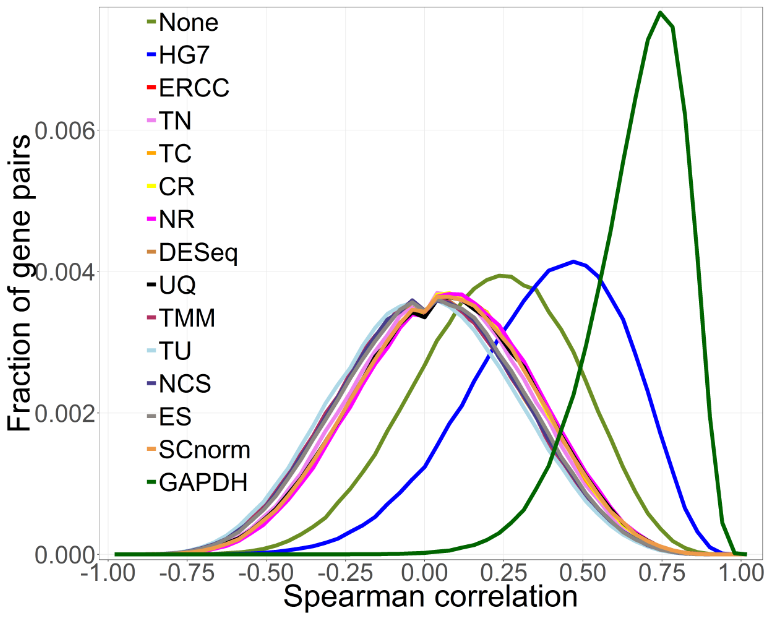


### 3.E Hierarchical clustering of normalization factors (single-cell data)

| tiff(file = "scRNA663_hc.tif", res=300, compression = "lzw",width=(2360*4.17),height=(1960*4.17));  plotHC(scRNA663_factors, method="spearman", mar=c(9,1,0,20))  dev.off(); |
| --- |

Results(**3E[1]**):


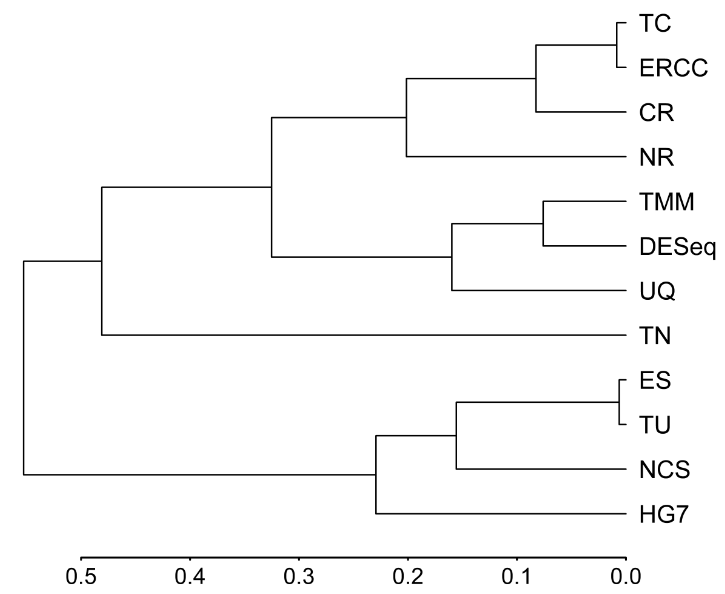


### 3.F Hierarchical clustering of normalization factors (bulk data)

| tiff(file = "bkRNA18_hc.tif", res=300, compression = "lzw",width=(2360*4.17),height=(1960*4.17));  plotHC(bkRNA18_factors, method="spearman", mar=c(9,1,0,20))  dev.off(); |
| --- |

Results(**3F[1]**):


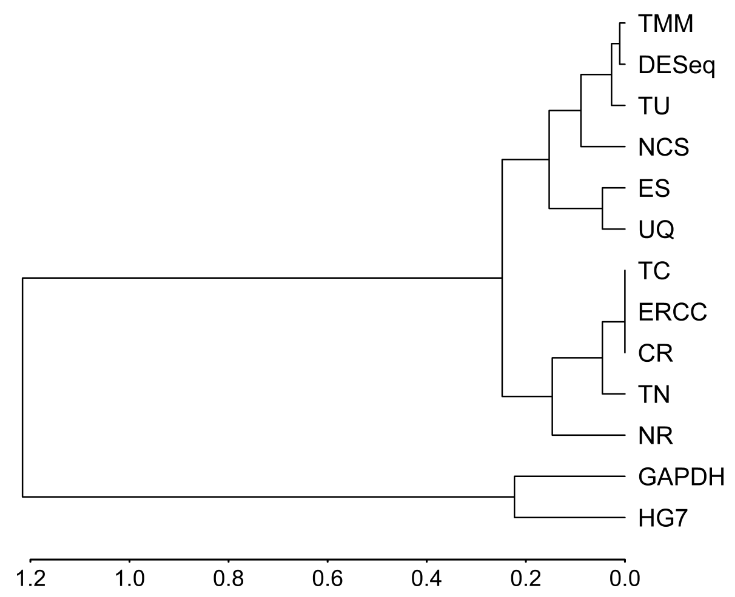


## 4 How to evaluate many users' methods

### 4.A For methods with normalization factors (single-cell data)

| # assume users' methods are named method1-9  # produce normalization factors named factor1-9  # run the same command line as **2.A**  scRNA663.AUCVCs <- gridAUCVC(data= scRNA663, dataType="sc", HG7= factor1, ERCC= factor2, TN= factor3, TC= factor4, CR= factor5, NR= factor6, DESeq= factor7, UQ= factor8, TMM= factor9, TU= 1, nonzeroRatios= c(0.2,0.3,0.4,0.5,0.6,0.7,0.8,0.9));  # Look at the maximum AUCVC for TU  scRNA663.AUCVCs1;  # Find the parameters used by TU when it achieved the maximum AUCVC in bestPara.txt bestPara <- read.table(file='bestPara.txt', header=TRUE);  bestPara;  # Using the parameters to produce the TU normalization factor  tu <- getFactors(data = scRNA663, method = "TU", pre_ratio=0.5, lower_trim=0.05, upper_trim=0.65);  # Produce mSCCs for 11 methods (Nonzero ratio=0.2)  scRNA663.cors1 <- gatherCors(data= scRNA663, cor_method="spearman", HG7= factor1, ERCC= factor2, TN= factor3, TC= factor4, CR= factor5, NR= factor6, DESeq= factor7, UQ= factor8, TMM= factor9, TU= tu, pre_ratio=0.5, lower_trim=0.05, upper_trim=0.65, rounds=1000000); |
| --- |

### 4.B For a general usage (single-cell data)

| # assume users' methods are named method1-9  # produce normalized gene expression matrics named matrix1-9  scRNA663.AUCVCs1 <- gridAUCVC4Matrices(None= scRNA663, m1 = matrix1, m2 = matrix2, m3 = matrix3, m4 = matrix4, m5 = matrix5, m6 = matrix6, m7 = matrix7, m8 = matrix8, m9 = matrix9, nonzeroRatios= c(0.2,0.3,0.4,0.5,0.6,0.7,0.8,0.9));  # Grid of non-zero ratios to produce AUCVCs for TU and **it is time-consuming**  scRNA663.AUCVCs2 <- gridAUCVC(data= scRNA663, dataType="sc", TU= 1, nonzeroRatios= c(0.2,0.3,0.4,0.5,0.6,0.7,0.8,0.9));  # Look at the maximum AUCVC for TU  scRNA663.AUCVCs2;  # Find the parameters used by TU when it achieved the maximum AUCVC in bestPara.txt  bestPara <- read.table(file='bestPara.txt', header=TRUE);  bestPara;  # Using the parameters to produce the TU normalization factor  tu <- getFactors(data = scRNA663, method = "TU", pre_ratio=0.5, lower_trim=0.05, upper_trim=0.65);  # Produce the TU-normalized gene expression matrix  TU_sc.matrix <- getNormMatrix(data = scRNA663, norm.factors = tu);  # Produce mSCCs for 11 methods (Nonzero ratio=0.2)  scRNA663.cors <- gatherCors4Matrices(None= scRNA663, m1 = matrix1, m2 = matrix2, m3 = matrix3, m4 = matrix4, m5 = matrix5, m6 = matrix6, m7 = matrix7, m8 = matrix8, m9 = matrix9, TU = TU_sc.matrix, raw_matrix= scRNA663, cor_method="spearman", pre_ratio=0.5, lower_trim=0.05, upper_trim=0.65, rounds=1000000); |
| --- |

## 5 Using other performance metrics

Two metrics are named as mean squared median relative log-expression (RLE_MED) and variance of inter-quartile range (IQR) of RLE (RLE_IQR). The evaluation results of three groups (particularly TU and ES) by RLE_MED were consistent with those by mSCC and by AUCVC using both scRNA-seq and bulk RNA-seq data. However, the evaluation results by RLE_IQR were not consistent with those by mSCC and by AUCVC.


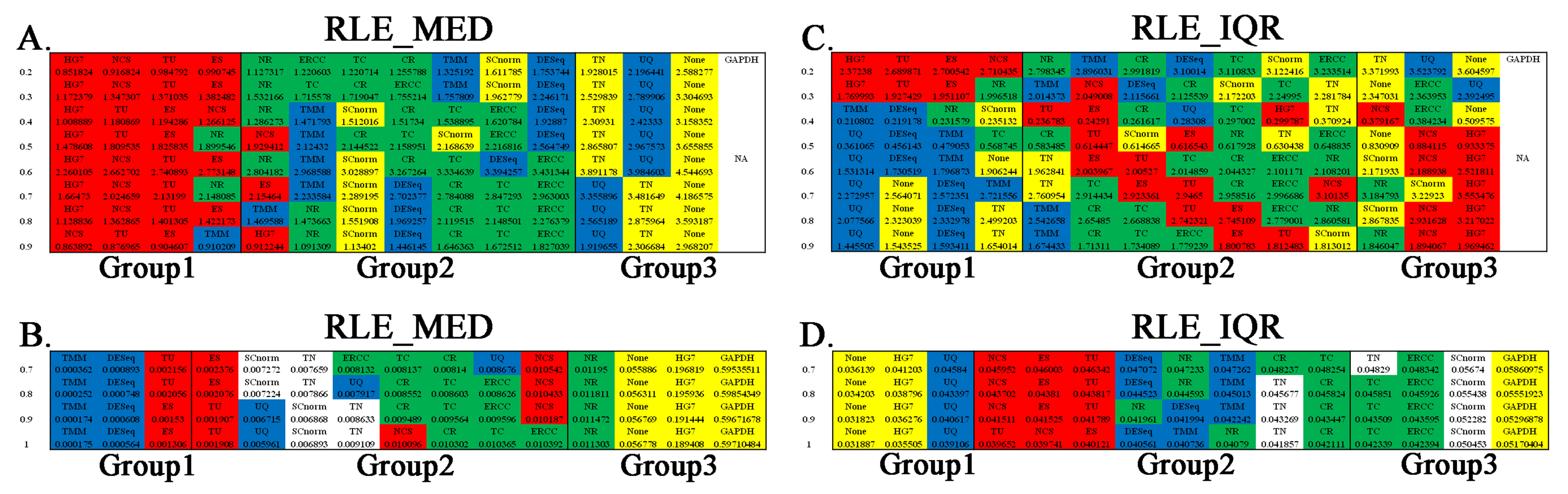


The non-zero ratio cutoffs from 0.2 to 0.9 for scRNA663 and from 0.7 to 1 for bkRNA18 were used to produce RLE_MEDs and RLE_IQRs. All the normalization methods were classified into three groups based on their RLE_MED values sorted in ascending order (from the best to the poorest) using one scRNA-seq dataset scRNA663 (**A**) and one bulk RNA-seq dataset bkRNA18 (**B**). These methods were also classified into three groups based on their RLE_IQR values sorted in ascending order (from the best to the poorest) using one scRNA-seq dataset scRNA663 (**C**) and one bulk RNA-seq dataset bkRNA18 (**D**). GAPDH is not applicable to scRNA-seq data due to zero counts of GAPDH present in many samples. All the numbers are accurate to two decimal places, the marginal differences are reflected by their orders. The raw gene expression data (None) was also used to produce RLE_MEDs and RLE_IQRs for comparison. After further normalization, RLE is identical to DESeq and presented as DESeq (RLE) or DESeq* in this study.

## 5 Package versions and FAQ


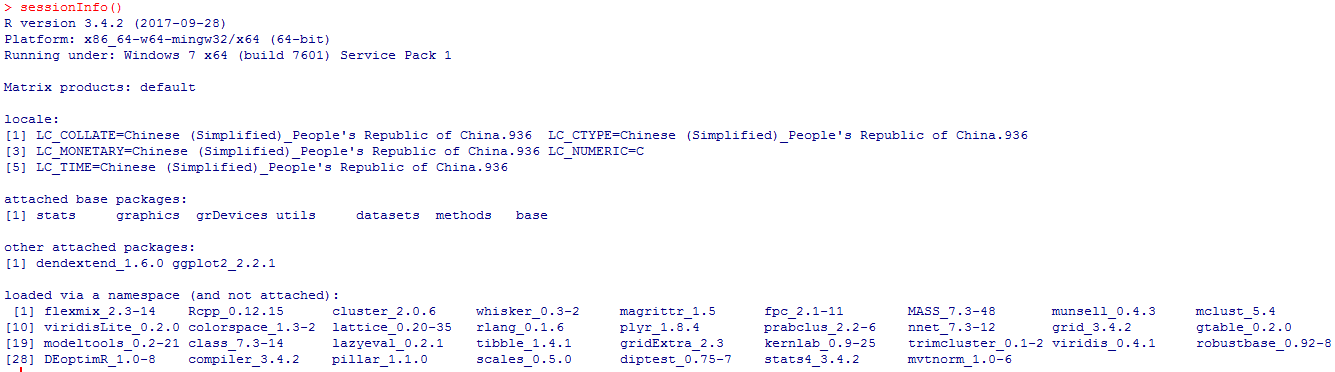

Supplement: FILE S1 — How to use NormExpression. [file Data_Sheet_1.doc]
